# Determination of Nitrogen Metabolism-Related Prognostic Signatures for Forecasting Bladder Cancer Prognosis

**DOI:** 10.2174/0118715303371907250514054016

**Published:** 2025-05-16

**Authors:** Hongtao Cheng, Yuhong Li, Shuyu Shen

**Affiliations:** 1 Department of Urology, Shulan (Hangzhou) Hospital, Shulan International Medical College, Zhejiang Shuren University, Hangzhou, 310022, China;; 2 Department of Anesthesiology, Shulan (Hangzhou) Hospital, Shulan International Medical College, Zhejiang Shuren University, Hangzhou, 310022, China;; 3 Department of Orthopedics, Hangzhou Xixi Hospital, Hangzhou, 310023, China

**Keywords:** Bladder cancer, nitrogen metabolism, machine learning, prognosis, immune infiltration, drug prediction

## Abstract

**Background:**

Bladder cancer is one of the major health threats worldwide, and aberrant regulation of nitrogen metabolism is closely related to its development. Understanding the role of nitrogen metabolism-related genes in BC is pivotal for the development of new therapeutic strategies and prognostic assessment.

**Aims and Objectives:**

This study aimed to explore the prognostic factors associated with nitrogen metabolism in bladder cancer (BC) and to construct a prognostic model.

**Methods:**

Differential expression gene analysis was performed to identify genes associated with nitrogen metabolism by analyzing mRNA expression data from BC patients. The prognostic relationship between these genes and BC patients was analyzed using univariate Cox regression. One hundred one combinatorial machine learning methods were applied for feature selection, and key prognostic genes were identified based on the method with the highest combined score. Immunocyte infiltration analysis was carried out to assess the tumor microenvironmental characteristics of patients in different risk groups.

**Results:**

Twenty-five genes significantly associated with prognosis were identified from nitrogen metabolism-related genes. Twenty-three most prognostically predictive signature genes were screened under feature screening with multiple machine-learning models. Immune cell infiltration analysis showed that patients in the high-risk group had significantly different immune cell infiltration, suggesting that these genes may influence BC progression by regulating immune escape mechanisms. These results provide new biomarkers and potential therapeutic targets for precision treatment and prognostic assessment of BC.

**Discussion:**

The findings suggest that nitrogen metabolism-related genes play a key role in the prognosis of bladder cancer and may be involved in regulating the tumor immune microenvironment. Different immune environments were demonstrated in high and low Riskscore groups, implying that these genes may contribute to immune evasion and thus promote tumor progression. These observations are consistent with emerging evidence that emphasizes the interplay between metabolism and immunity during cancer development. By combining nitrogen metabolism with immune analysis, this study provides a new perspective for stratifying BC patients and identifying therapeutic targets.

**Conclusion:**

The expression patterns of nitrogen metabolism-related genes identified can be used as effective biomarkers for bladder cancer prognosis, providing a scientific basis for personalized treatment. Future studies can further explore the specific biological functions and mechanisms of action of these genes to promote more effective clinical applications.

## 1 INTRODUCTION

Bladder cancer is a pivotal public health problem globally. In 2020, there were approximately 573,000 newly diagnosed cases of bladder cancer and 213,000 deaths worldwide. The incidence and mortality rates of bladder cancer vary in different regions and are usually higher in men than in women. Known risk factors include smoking, occupational exposure to harmful chemicals, and drinking contaminated water [1]. Recent advances in the treatment of bladder cancer have significantly changed the choice of treatment options. Targeted therapies and immunotherapy have become more pivotal, especially with the introduction of drugs, such as pembrolizumab, avelumab, and enfortumab vedotin, which have shown promise in improving survival in advanced cases [2-4]. The combination of erlotinib and pembrolizumab doubled survival rates over conventional chemotherapy in certain patient groups. Current clinical challenges in the treatment of bladder cancer include managing the high rates of recurrence and progression in non-muscle invasive bladder cancer (NMIBC), integrating genetic testing to customize treatment plans, and overcoming treatment resistance. In addition, the cost of new treatments, such as immunotherapy, remains a concern [5, 6]. However, there is still a large proportion of patients who are insensitive to immunotherapy and for whom there are currently no effective biomarkers [7]. Therefore, it is crucial to develop effective therapeutic and prognostic biomarkers.

Recent studies have reported that nitrogen metabolism plays a pivotal role in the development of bladder cancer, and in particular, there is growing interest in the role of N6-methyladenosine (m6A) modifications in the regulation of tumor cells. For example, YTHDF2, an m6A-reading protein, promotes bladder cancer progression by inhibiting RIG-I-mediated immune response, thus playing a key role in bladder cancer cell proliferation and tumor growth. Aberrant expression of YTHDF2 can regulate tumor immune escape through the degradation of RIG-I-associated mRNAs, which provides a new target for bladder cancer therapy [8]. In addition, the role of m6A in RNA metabolism includes influencing mRNA splicing, nucleation, translation initiation, and degradation, with METTL3 and METTL14 being pivotal m6A methyltransferases involved in regulating these processes. The study of these mechanisms not only increases our understanding of the molecular mechanisms of bladder cancer but also offers the possibility of developing new diagnostic and therapeutic strategies [9, 10]. Studies of nitrogen metabolism have also addressed its regulatory effects on the tumor microenvironment, including influencing the biological behavior of immune cells, which is particularly pivotal in cancer therapy. Thus, a deeper understanding of the role of nitrogen metabolism in bladder cancer could help in the development of therapeutic strategies targeting this metabolic pathway, potentially improving the targeting and efficacy of treatment [11].

In this study, a prognostic scoring model (Risk Score) was constructed and validated by integrating mRNA expression data and nitrogen metabolism-related genes of bladder cancer, which can effectively distinguish between high and low-risk groups of bladder cancer patients. Twenty-three key prognostic signature genes were screened by multiple machine learning methods and utilized to demonstrate good predictive performance in the training set and external validation set. In addition, the study provides an in-depth analysis of pathway activity, immune infiltration, and drug sensitivity in the high- and low-risk groups, providing potential biomarkers and therapeutic targets for personalized treatment of bladder cancer.

## 2 MATERIALS AND METHODS

### 2.1 Source of Dataset

The Cancer Genome Atlas database (TCGA, https://portal.gdc.cancer.gov/) was accessed, from which we retrieved the mRNA expression data of bladder cancer with the data type count. The dataset consisted of 19 normal samples and 414 tumor samples, and the clinical information was screened for survival time records with more than 30 days, which were used as the training set. We also downloaded the corresponding SNP data with the file name input .maf for subsequent gene variant analysis. Gene expression data of bladder cancer sample set GSE13507 and its related clinical information were obtained from the Gene Expression Omnibus database (GEO, https://www.ncbi.nlm.nih.gov/gds), which were used as a validation set. The platform annotation file for this dataset was GPL6102 to ensure proper interpretation and application of the data. To deeply investigate the role of nitrogen metabolism in bladder cancer, 881 genes related to nitrogen metabolism were downloaded from the GeneCards database (https://www.genecards.org/). This study adhered to the REMARK guidelines for transparent reporting of tumor marker prognostic studies [12].

### 2.2 Prognostic Modeling of Nitrogen Metabolism-Related Genes

The mRNA expression data of bladder cancer were differentially analyzed using the R package edgeR, and the screening criteria were set as absolute logarithmic fold change greater than 1 (|logFC| > 1) and hypothetical discovery rate less than 0.05 (FDR < 0.05) [12]. Using the upsetsplot package, differential genes were analyzed for intersection with genes related to nitrogen metabolism and screened for genes common to both [13]. Univariate Cox regression analysis was performed on the genes in the intersection set using the R package survival to further screen for genes that were significantly associated with prognosis (*p* < 0.01) [14]. To prevent model overfitting, we used a combination of machine learning methods, including randomForestSRC, glmnet, plsRcox, SuperPC, Gradient Boosting Machine (gbm), survivalsvm, and BART, resulting in a total of 101 feature selection methods [15-21]. The best feature selection method was finalized by comparing the C-index scores. Multivariate Cox regression models were constructed using the survival package and the screened feature genes to assess the association between these genes and the prognosis of bladder cancer patients. Forest plots were drawn using the survminer package to visualize the influence and statistical significance of each gene [22].

### 2.3 Prognostic Model Validation

Based on the expression levels of prognostic signature genes and multivariate Cox regression coefficients, we constructed an assessment model, Risk Score, for predicting the prognosis of BC patients. The principal component analysis (PCA) was performed on the modeled signature genes of the high- and low-risk groups using the R packages FactoMineR [23]. Based on the high and low-risk score groups, Kaplan-Meier (K-M) survival analysis was performed using the R package survival, and survival curves were plotted using the survminer package. This step was designed to assess the impact of risk score grouping on patient prognosis. ROC curve analysis was carried out using the R package timeROC, and AUC values for 1-, 3- and 5-year overall survival (OS) were calculated to assess the predictive accuracy of the model [24]. Finally, to verify the robustness of the model, the model was validated in the external validation set GSE13507. Moreover, to explore the distribution of risk score in molecular subtypes of bladder cancer, we extracted the genes characterizing the six molecular subtypes, namely Luminal Papillary (LumP), Luminal Non-Specified (LumNS), Luminal Unstable (LumU), Stroma-rich, Basal/Squamous (Ba/Sq), and Neuroendocrine-like (NE-like), from the study by Kamoun *et al*., to subtype patients and observe the distribution of risk score among them [25].

### 2.4 Pathway Analysis

KEGG pathway enrichment analysis was performed on the high- and low-risk groups using GSEA software to screen out biologically significant pathways. A hypothetical discovery rate (FDR) of less than 0.25 was set as the significance threshold to ensure the reliability of the results.

### 2.5 Immune Infiltration Analysis

Samples from high and low-risk groups were evaluated using the ESTIMATE package to calculate the purity of immune, stromal, and tumor cells, as well as the combined ESTIMATE score [26]. Analysis was performed based on the ssGSEA algorithm implemented in the GSVA package using a database containing 29 immune-related gene sets [27].

### 2.6 Mutation Characterization

Using the R package maftools, we performed a detailed mutation site analysis of high-frequency mutated genes in the high- and low-risk groups [28]. This included visualization of the distribution of genes with high mutation frequencies in different risk groups to visualize the mutations in these genes. We paid particular attention to the co-mutation and mutually exclusive relationships between these high-frequency mutated genes. This helps us understand the unique or shared genetic variation pathways that may exist in different risk groups.

### 2.7 Drug Sensitivity Analysis

To improve the accuracy of predicting response to breast cancer treatment, we utilized the Genomics of Drug Sensitivity in Cancer database (GDSC, https://www.cancerrxgene.org/). Using the R package oncoPredict, we downloaded gene expression profiles and corresponding drug response information from the GDSC2 database [29]. These data were used to construct a ridge regression-based model designed to predict the half-maximal inhibitory concentration (IC50) for all drugs in breast cancer patients. Based on patient subgroups, we performed t-test analyses of drug response to explore differences in response to specific chemotherapy candidate compounds among patients in different risk score groups. Particular attention was paid to compounds that may be more effective in breast cancer patients in subgroups with high (or low) iron metabolism and nitrogen metabolism. Violin plots were created using the ggpubr package to visualize the differences in sensitivity to various chemotherapeutic agents in patients of different risk score groups [30]. This helps us to understand more clearly the association between drug sensitivity and patient risk class.

### 2.8 Independence Validation and Nomogram Construction for Risk Score

In order to comprehensively assess the independent role of risk score in prognostic analysis, univariate and multivariate Cox analyses were performed to validate the clinical independence of risk score using the R package survival combining clinical factors and risk score. Nomograms were constructed using the rms package combining clinical characteristics and risk scores, and 1-, 3-, and 5-year calibration curves were plotted to assess the predictive accuracy of the column charts [31].

## 3 RESULTS

### 3.1 Prognostic Modeling of Nitrogen Metabolism-Related Genes

**Fig. (2):**
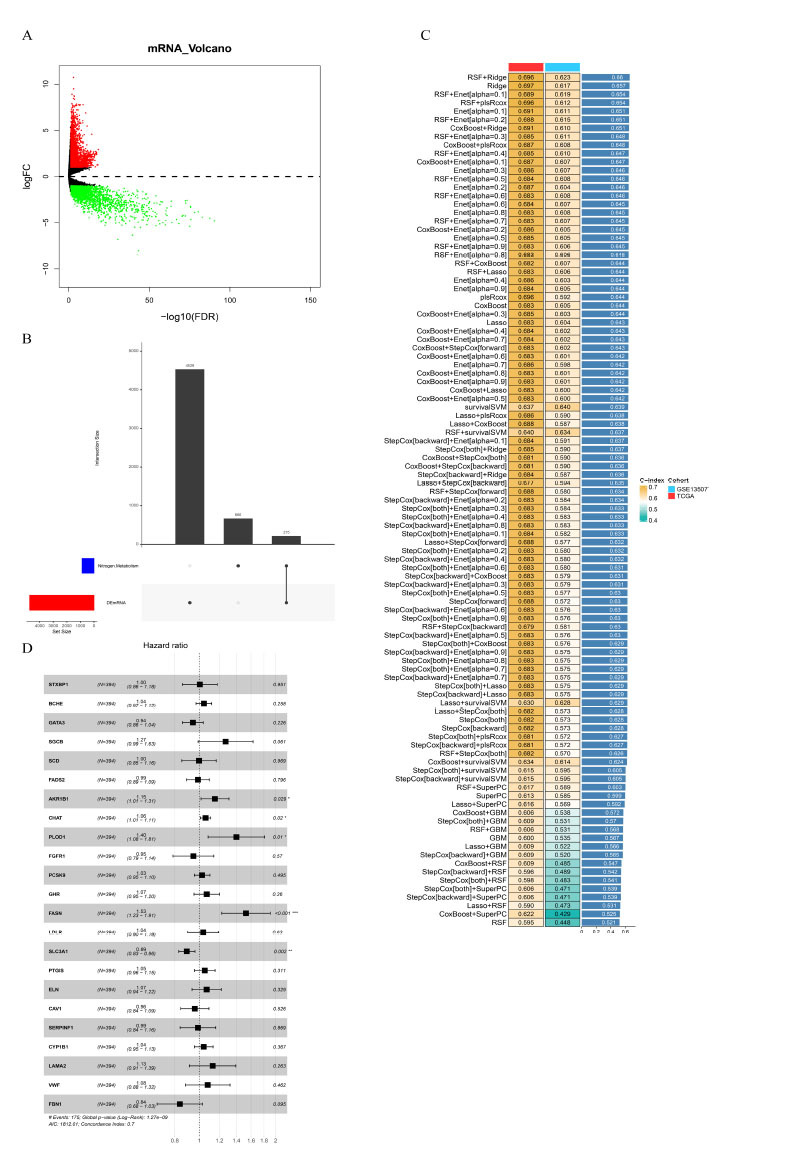
Prognostic model construction of nitrogen metabolism-related genes. (**A**) The volcano plot demonstrates the differentially expressed mRNAs. (**B**) The UpSet plot demonstrates the overlapping genes in differentially expressed mRNAs and nitrogen metabolism-related genes. (**C**) C-Index scores of 101 machine learning combinatorial model feature selection results. (**D**) Forest plot of multivariate Cox analysis of 23 prognosis-related feature genes.

The flow chart of this study is shown in Fig. (**1**). In the differential analysis, we identified 4744 differentially expressed mRNAs (Fig. **2A**). By intersecting these differential genes with 881 genes involved in nitrogen metabolism in the intersection analysis, we finally obtained 215 genes that could be used in subsequent studies (Fig. **2B**). Using TCGA bladder cancer gene expression data as a training set, univariate Cox regression analysis screened 25 genes that were significantly associated with patient prognosis. Among these genes, we used 101 different combinatorial machine learning methods to select features for 20 genes, among which the combination method of RSF plus ridge regression had the highest combined score and screened 23 candidate feature genes (Fig. **2C**). Subsequently, these 23 candidate genes were further validated by multivariate Cox regression analysis and finally identified as key features for constructing prognostic risk models for bladder cancer patients (Fig. **2D**).

### 3.2 Prognostic Model Validation

**Fig. (3):**
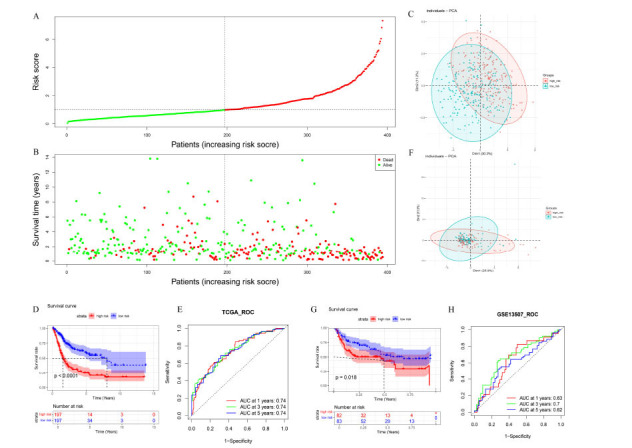
Prognostic model validation. (**A**) Distribution of samples in the high and low Riskscore groups in the TCGA-BLCA dataset, grouped according to the median Riskscore value. (**B**) Distribution of survival status of patients in the high and low Riskscore groups in the TCGA-BLCA dataset. The red dots in the graph are samples that died and the green dots are samples that survived. (**C**) Plot of PCA results for categorizing samples based on Riskscore consisting of 23 characterized genes in TCGA-BLCA dataset. (**D**) K-M curves of patients with high and low Riskscore groupings in TCGA-BLCA dataset. (**E**) ROC curves for Riskscore prediction of 1-, 3-, and 5-year survival in BC patients in the TCGA-BLCA dataset. (**F**) Plot of PCA results for categorizing samples based on Riskscore consisting of 23 characterized genes in GSE13507 dataset. (**G**) K-M curves of patients with high and low Riskscore groupings in GSE13507 dataset. (**H**) ROC curves for Riskscore prediction of 1-, 3-, and 5-year survival in BC patients in the GSE13507 dataset.

In the TCGA-BLCA dataset, the prognostic model, risk score, for BC was constructed based on the expression levels of 23 prognosis-related genes and multivariate Cox coefficients. The samples were categorized into the high-risk score group and the low-risk score group according to the median risk score value (Fig. **3A**). More cases died in the high-risk score group (Fig. **3B**). The PCA results also showed that the samples could be well differentiated according to the grouping of the 23 characterized genes (Fig. **3C**). In the training set, it was clearly observed that patients in the high-risk score group exhibited worse survival (Fig. **3D**). The ROC curves showed that the AUC values of a risk score for predicting 1-, 3-, and 5-year survival of BC patients were all 0.74, indicating that the risk score has good predictive performance (Fig. **3E**). In the GSE13507 cohort, the PCA results showed some degree of differentiation in the high and low-risk score groupings with switching, but there was overlap (Fig. **3F**). Similarly, patients in the low-risk score group showed better prognostic characteristics (Fig. **3G**). To verify the robustness of the risk score, external validation was performed on the validation set GSE13507 dataset. The ROC curves showed that the AUC values of risk score for predicting the 1-year, 3-year, and 5-year survival rates of BC patients were 0.63, 0.7, and 0.62, respectively (Fig. **3H**). Additionally, the distribution of risk scores in patients with six subtypes (LumP, LumNS, LumU, Stroma-rich, Ba/Sq, and NE-like) was assessed. The NE-like patients exhibited the highest levels of risk score. High-risk score patients mapped more to the Ba/Sq subtype, and low-risk score patients mapped more to the LumP subtype (Supplementary Fig. **1**). Overall, the risk score showed good robustness both in the training set and in the external validation set, indicating that the risk score is a reliable prognostic tool.

### 3.3 Differences in Pathways Immune Infiltration in High and Low-Risk Score Groups

**Fig. (4):**
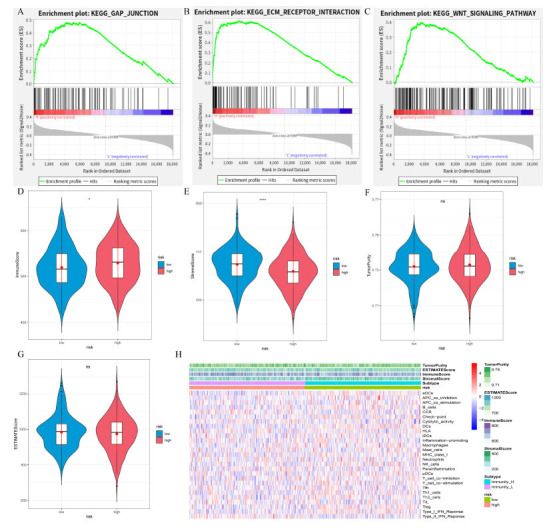
Pathway differences and immune infiltration differences in the high- and low-risk score groups. (**A-C**) KEGG pathways with differences in high- and low-risk score groups. (**A**) gap junction. (**B**) ecm_receptor_interaction. (**C**) wnt signaling pathway. (**D-G**) Results of estimate immune infiltration analysis in the high and low-risk score groups. (**D**) immune scores. (**E**) Stromal scores. (**F**) Tumor purity. (**G**) Estimate scores. (**H**) Heatmap of 29 immune cell infiltration levels in the high- and low-risk score groups.

KEGG pathways with differences were identified in the high and low-risk score groups. The results showed that the activities of GAP JUNCTION, ECM_RECEPTOR_INTERACTION, and WNT SIGNALING PATHWAY were activated in the high-risk score group (Figs. **4A**-**C**). Subsequently, we also compared the immune scores of patients in the two groups. The results showed that patients in the high-risk score group exhibited higher immune scores, and patients in the low-risk score group exhibited higher stromal scores (Figs **4D**-**E**). The differences in estimate scores and tumor purity were not significant in the two groups (Figs. **4F**-**G**). According to the ssGSEA results of the 29 immune gene set, immune infiltration scores were higher in the high-risk score group, suggesting that patients with high-risk scores may exhibit an immune “hot” phenotype (Fig. **4H**).

### 3.4 Mutation Characterization and Drug Sensitivity Analysis in High- and Low-Risk Score Groups

**Fig. (5):**
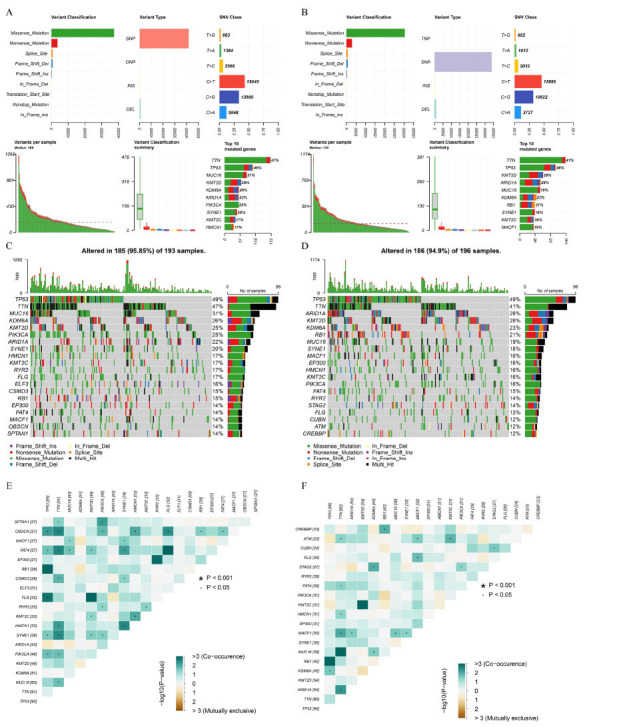
Characterization of mutations in the high- and low-risk score groups. (**A**) Statistical graph of high-frequency mutated genes, mutation sites, and mutation types in the low-risk score group. (**B**) Statistical graph of high-frequency mutated genes, mutation sites, and mutation types in the high-risk score group. (**C**) Waterfall of top 20 mutated genes in the low-risk score group. (**D**) Waterfall plot of top 20 mutated genes in the high-risk score group. (**F**) Mutation frequency of top 20 genes in the high-risk score group with co-mutations and mutually exclusive mutations.

**Fig. (6):**
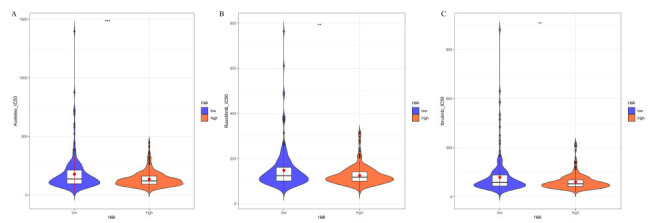
Treatment sensitivity analysis of acetalax, ruxolitinib, and ibrutinib in the high- and low-risk score groups. (**A**) IC50 of acetalax in high- and low-risk score groups. (**B**) IC50 of ruxolitinib in high- and low-risk score groups. (**C**) IC50 of ibrutinib in high- and low-risk score groups.

The mutation characteristics in the high- and low-risk score groups were also analyzed. The proportion of Missense_Mutation and SNPs was higher in the low-risk score group. TTN, TP53, and MUC16 were the top 3 genes with mutation frequency in the low-risk score group (Figs. **5A** and **C**). Similarly, the proportion of Missense_Mutation and SNPs was higher in the high-risk score group. TTN, TP53, and KMT2D were the top 3 genes with mutation frequency in the high-risk score group (Figs. **5B** and **D**). Co-mutation and mutually exclusive mutation phenomena were higher in the low-risk score group (Figs. **5E**-**F**). In addition, in the high- and low-risk score groups, we also evaluated the therapeutic sensitivity of acetalax, ruxolitinib, and ibrutinib. The IC50 values of the three drugs were lower in the high-risk score group, indicating that they had better treatment sensitivity in the high-risk score group (Figs. **6A**-**C**).

### 3.5 Clinical Independence Validation of Risk Score and Nomogram Predicting BC Survival

**Fig. (7):**
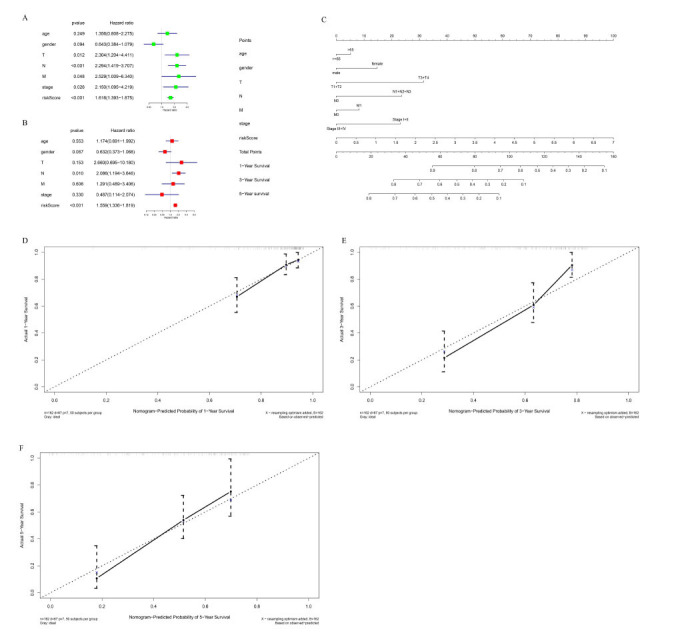
Nomogram of risk score for clinical independence validation and prediction of bc survival. (**A**) Results of univariate Cox regression analysis based on TCGA-BLCA training set data combined with clinical information and risk scores. (**B**) Results of Multivariate Cox regression analysis based on TCGA-BLCA training set data combined with clinical information and risk scores. (**C**) Nomogram constructed based on TCGA-BLCA training set data. (**D-F**) Calibration curves at 1, 3, and 5 years.

Univariate Cox analysis showed that T, N, M, stage, and risk score were clinically independent prognostic factors for BC (Fig. **7A**). Multivariate Cox analysis showed that N stage and risk score were clinically independent prognostic factors for BC (Fig. **7B**). Overall, the risk score model constructed based on the 23 characterized genes was a prognostic factor for BC. Based on risk score and clinical factors, we constructed nomograms for predicting 1-, 3-, and 5-year survival of BC patients (Fig. **7C**). The calibration curves showed that the prediction curves for 1-, 3-, and 5-year survival rates predicted based on the nomogram fitted well with the actual observed survival curves (Fig. **7D**-**F**). These results indicate good prognostic prediction tools.

## 4 DISCUSSION

Recent advances in the diagnosis and treatment of bladder cancer have been impressive, yet survival in bladder cancer remains suboptimal due to persistent challenges, such as metastasis and recurrence. The link between nitrogen metabolism and bladder cancer prognosis is becoming clearer in recent studies. A study identified metabolite markers in bladder cancer patients identified by mass spectrometry and nuclear magnetic resonance techniques, which reflect themolecular features of bladder cancer development [32]. In addition, changes in metabolites not only indicate disease status but may also be closely related to patient prognosis. For example, some studies have found that specific metabolite changes correlate with disease severity and prognosis by analyzing urine and serum samples from bladder cancer patients [33]. The application of such metabolite markers may be useful for early diagnosis and monitoring of treatment effects, which, in turn, may improve patient management strategies. In this study, we successfully constructed a prognostic model for bladder cancer based on genes related to nitrogen metabolism, and the results showed that the risk score exhibited excellent robustness in predicting the prognosis of BC.

In this study, by combining multiple machine learning methods, we identified 23 nitrogen metabolism genes that were significantly associated with bladder cancer prognosis. Most of these genes were found to be associated with cancer progression or prognosis. STXBP1 was found to be specifically dysregulated in non-mutated muscle-invasive tumors [34]. BCHE was identified as a prognostic biomarker in endometrial cancer and was found to be correlated with immunity [35]. SGCB may affect the clinical prognostic status of patients with hepatocellular carcinoma as well as their sensitivity to immunotherapeutic agents [36]. SCD is linked to the upregulation of stearoyl-CoA desaturase, and its inhibition can lead to a balance between unsaturated and saturated fatty acids, a phenomenon that may retard tumor tissue growth [37]. Upregulation of SCD accelerates lipid metabolism activity and cell invasiveness in tumor cells. Similarly, aberrant upregulation of FADS2 was found to increase tumor cell invasiveness, and SCD1/FADS2-associated inhibitors slowed cancer progression [38]. Machine learning algorithms are a common tool in identifying biomarkers. Deep algorithms have been extended, and the study of cancer prognosis and its diagnosis has reached new heights [39, 40]. According to our findings, the risk score model composed of these markers showed excellent predictive performance in multiple datasets. Patients with low-risk scores presented a better survival advantage. In addition, the risk score model can also be used to assess immune infiltration as well as therapeutic sensitivity to chemotherapeutic agents in bladder cancer patients. These findings suggest that risk score may be a good prognostic assessment tool for bladder cancer.

A study by Kamoun **et al*.* identified six dominant molecular subtypes in bladder cancer, namely LumP, LumNS, LumU, Stroma-rich, Ba/Sq, and NE-like. A NE-like subtype is highly aggressive, proliferative, poorly responsive to immunotherapy, and has a poor prognosis. Our results demonstrated the highest risk score among NE-like subtypes, with a high-risk score found in the Ba/Sq subtype and a low-risk score in the LumP subtype. In the risk score model of this study, the highest scores indicated that the characteristic gene expression pattern of these samples predicts that they have the worst survival prognosis, which is highly consistent with the clinical presentation of the NE-like subtypes. Ba/Sq is a high-risk subtype characterised by the expression of stemness markers and associated with epithelial-mesenchymal transition (EMT), TGF-β signalling, and immune escape mechanisms. While it is typically chemosensitive, it exhibits poor response to immunotherapy, correlating with a high-risk score. LumP is a subtype with an excellent prognosis, characterised by a high degree of differentiation, typically good response to treatment, and a low tumor proliferation index. This profile suggests that a low-risk score correlates with a transcriptional profile associated with favourable outcomes, aligning with the objectives of risk score modelling. Consequently, this indicates that the risk score possesses strong molecular classification relevance and biological explanatory power [25]. Recent studies have developed several bladder cancer prognostic models, such as the A score model based on apoptosis-related genes and the 11-gene signature model based on lipid metabolism. The A score model has demonstrated strong predictive power for immunotherapy response and has been validated in several clinical cohorts [41]. Lipid metabolism modeling has helped to gain insight into fatty acid-related signaling and immune microenvironment characterization [42]. In contrast to these studies, to the best of our knowledge, our model is the first to focus on nitrogen metabolism, a metabolic pathway that intersects with immune function, hypoxia adaptation, and tumor cell proliferation. In addition, the comprehensive machine learning screening process employed in this study ensures that the final 23 gene features are not only of prognostic value but also biologically relevant and mechanistically informative.

Our study still has limitations. First, this study analyzed data in TCGA and GEO datasets with relatively small sample sizes, and a larger cohort is still needed for external validation in the future. Second, although we identified 23 biomarkers that can be used to predict the prognosis of bladder cancer, we still need more clinical information, such as measuring their expression information when collecting patient samples to optimize risk scores. 

## CONCLUSION

In this study, we systematically constructed and validated a prognostically relevant feature-scoring model by focusing on the potential role of nitrogen metabolism in bladder cancer, reflecting the deep association between metabolic regulation and immune microenvironment remodeling. By integrating multiple machine learning methods with multidimensional bioinformatics analysis, this study not only deepens our understanding of the relationship between tumor metabolic heterogeneity and clinical outcomes but also provides a theoretical basis for individualized risk assessment and targeted intervention in the context of precision medicine. In the future, the combination of multi-omics data and functional experiments will help to further reveal the specific mechanism of nitrogen metabolism-driven tumor progression and promote its translational application in diagnostic and therapeutic integration.

## Figures and Tables

**Fig. (1):**
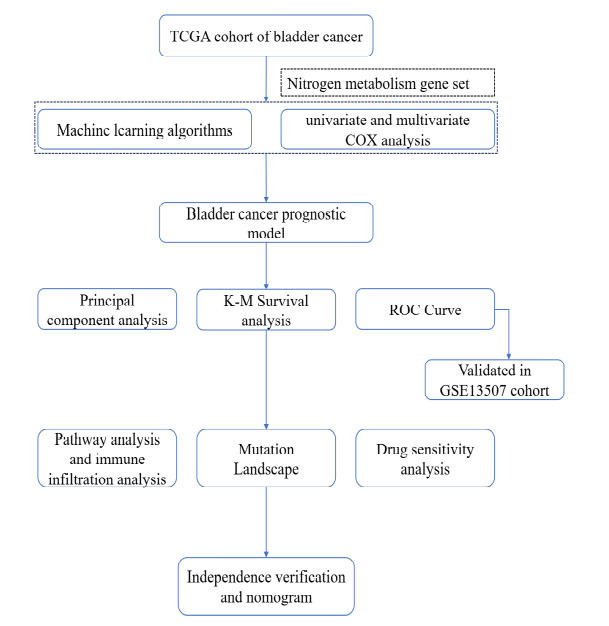
The flowchart of this study.

## Data Availability

The datasets generated and/or analyzed during the current study are available in the GSE13507 repository (https://www.ncbi.nlm.nih.gov/geo/query/acc.cgi?acc= GSE13507).

## LIST OF ABBREVIATION

NMIBC: Non-muscle Invasive Bladder Cancer
m6A: N6-methyladenosine
TCGA: The Cancer Genome Atlas Database
GEO: Gene Expression Omnibus
Gbm: Gradient Boosting Machine
PCA: Principal Component Analysis
K-M: Kaplan-Meier
OS: Overall Survival
FDR: Hypothetical Discovery Rate
GDSC: Genomics of Drug Sensitivity in Cancer
