# Supplementary material for: Determination of Nitrogen Metabolism-Related Prognostic Signatures for Forecasting Bladder Cancer Prognosis
Source: Endocr Metab Immune Disord Drug Targets. 2025 May 16;26:E18715303371907. doi: 10.2174/0118715303371907250514054016 (PMC13334269; doi:10.2174/0118715303371907250514054016)
Supplement: Supplementary file 1 [file EMIDDT-26-E18715303371907_SD1.pdf]

Supplementary Material

Determination of Nitrogen Metabolism-Related Prognostic Signatures for Forecasting Bladder Cancer Prognosis

Hongtao Cheng[1,*], Yuhong Li[2] and Shuyu Shen[3]

[1]Department of Urology, Shulan (Hangzhou) Hospital, Shulan International Medical College, Zhejiang Shuren University, Hangzhou, 310022, China; [2]Department of Anesthesiology, Shulan (Hangzhou) Hospital, Shulan International Medical College, Zhejiang Shuren University, Hangzhou, 310022, China; [3]Department of Orthopedics, Hangzhou Xixi Hospital, Hangzhou, 310023, China

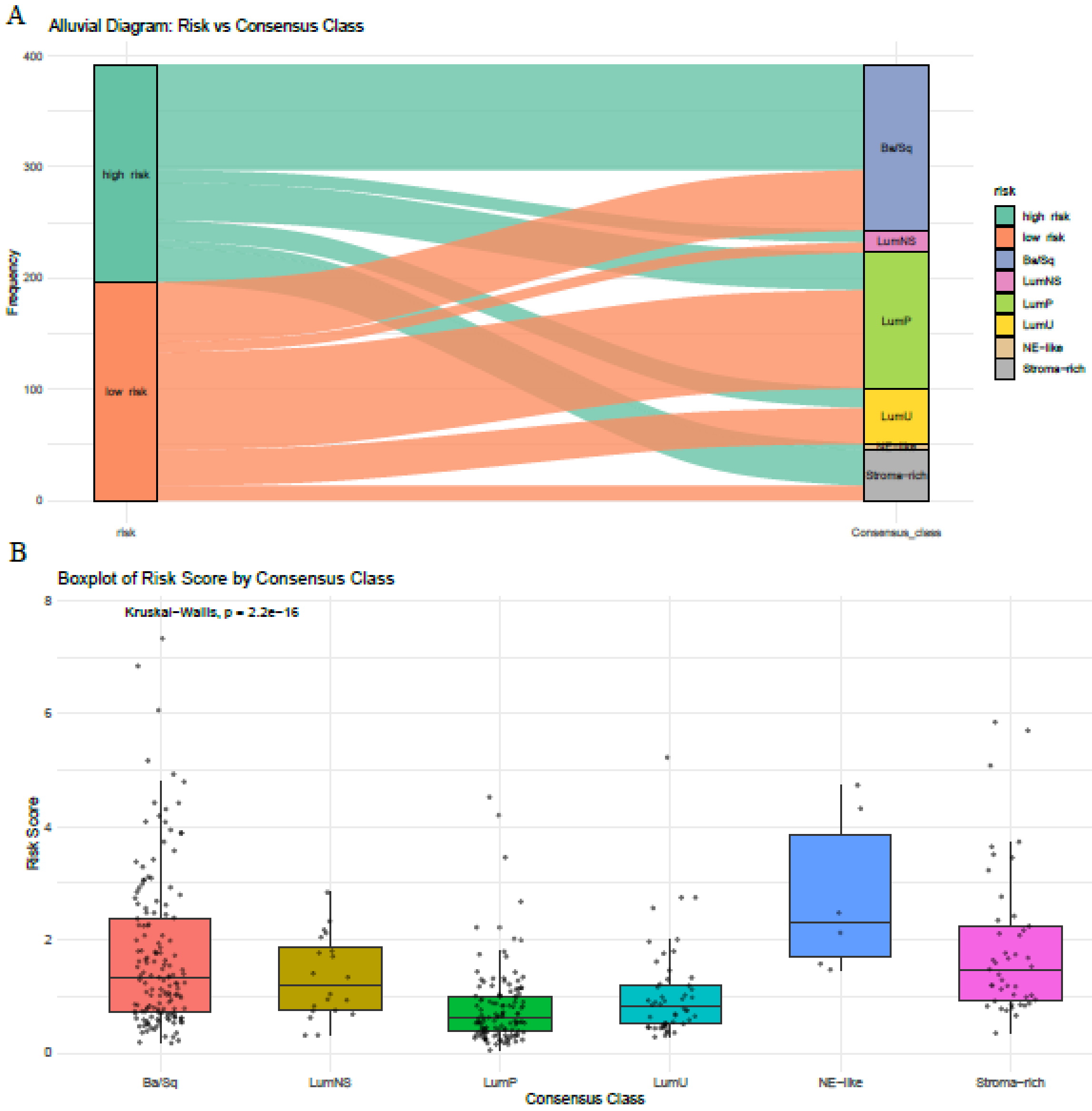

Supplemental figure 1: Distribution of Riskscores in six subtypes of bladder cancer. (**A**) Sankey diagram mapping between high and low riskcore patients and six subtypes of patients. **(B**) Statistical analysis of Riskscore in six subtypes of patients.
